# Mutant Cullin causes cardiovascular compromise

**DOI:** 10.15252/emmm.201505620

**Published:** 2015-08-20

**Authors:** Friedrich C Luft

**Affiliations:** Experimental and Clinical Research Center, a cooperation between the Max-Delbrück Center for Molecular Medicine and the Charité Medical FacultyBerlin, Germany

## Abstract

Mendelian hypertension is rare; however, Mendelian syndromes have taught us an amazing amount about mechanisms of distal sodium and chloride reabsorption, as well as how systemic hypertension might come about. In this issue of *EMBO Molecular Medicine*, Schumacher *et al* (2015) present a mouse model of the Cullin-3 (CUL3Δ403–459) mutation, which causes a form of pseudohypoaldosteronism type-2 (PHA-2). CUL3 is involved in ubiquitination. Surprising is the severity of the hypertension, which may be explained in part on the basis of CUL3 actions in vascular cells. The findings underscore the role of “cleanup” in the maintenance of normal physiology.

See also: **F-R Schumacher *et al*** (October 2015)

An apparent state of renal tubular unresponsiveness or resistance to the action of aldosterone characterizes a heterogeneous group of disordered electrolyte metabolism termed pseudohypoaldosteronism (PHA). PHA1 is characterized by sodium wasting, hyperkalemia, as well as hypotension and will not concern us further here. PHA2 features sodium retention, hyperkalemia, mild hyperchloremic metabolic acidosis, and hypertension. Mutations of the mammalian with-no-lysine kinases (WNK1 and WNK4) in humans cause PHA2 (Huang & Cheng, 2015). The importance of this rare and easily treated Mendelian hypertension lies in the pathways uncovered by this research (Sohara & Uchida, 2015). In the kidney, the WNK pathways, via protein odd-skipped-related-1 (OSR1) and STE20/SPS1-related proline/alanine-rich kinase (SPAK) (Rafiqi *et al*, 2010), are responsible for sodium-chloride cotransporter (NCC), sodium-potassium-2chloride transporter (NKCC), the renal epithelial sodium channel (ENaC), and renal outer-medullary potassium channel (ROMK) regulation (Huang & Cheng, 2015; Sohara & Uchida, 2015). ENaC subunits are ubiquitinated by the neural precursor cell expressed developmentally downregulated protein-4 (NEDD4). Failure of this process can also result in salt-sensitive hypertension through hyperactivity of ENaC in the distal nephron.

paracellular pathway is physiologically regulated through the ubiquitination pathway, and its deregulation may lead to diseases of electrolyte and blood pressure imbalances

Pseudohypoaldosteronism type IIE (PHA2E) is caused by heterozygous mutation in the *CUL3* gene on chromosome 2q36. Boyden *et al* used exome sequencing to identify mutations in Kelch-like 3 (*KLHL3*) or *CUL3* in PHA2 patients from 41 unrelated families (Boyden *et al*, 2012). *KLHL3* mutations were either recessive or dominant, whereas *CUL3* mutations were dominant and predominantly *de novo*. The Kelch motif is composed of about 50 amino acid residues forming a four-stranded beta-sheet “blade”. The sequence motif commonly exists as six or eight copies per protein forming a circular solenoid structure called a beta-propeller domain. The authors suggested that CUL3 and the BTB-domain-containing KLHL3 proteins are components of cullin-really interesting new gene (RING) E3 ligase complexes that ubiquitinate substrates bound to Kelch propeller domains (Boyden *et al*, 2012). Ohta *et al* subsequently immunoprecipitated KLHL3 and found that KLHL3 was strongly associated with WNK isoforms and CUL3, but not with other components of the pathway regulating NCC (Ohta *et al*, 2013). Furthermore, the *KLHL3* disease mutations that they analyzed inhibited binding to WNK1 or CUL3. They next mapped the KLHL3 interaction site in WNK1 to a non-catalytic region. Their results suggested that the CUL3-KLHL3 E3 ligase complex regulates blood pressure via interaction with, and ubiquitination of WNK isoforms. Their study revealed how mutations disrupting the ability of an E3 ligase to interact with and ubiquitinate a critical cellular substrate such as WNK isoforms can trigger hypertension.

Shibata *et al* (2013) next presented findings demonstrating that CUL3-RING ligases containing KLHL3 target ubiquitination of WNK4 and thereby regulate WNK4 levels, which in turn control the levels of ROMK. These findings revealed a specific role for CUL3 and KLHL3 in electrolyte homeostasis and provided a molecular explanation for the effects of disease-causing mutations in both *KLHL3* and *WNK4*. McCormick *et al* (2014) then demonstrated that a PHA2E-causing CUL3 mutant (CUL3Δ403–459) not only retained the ability to bind and ubiquitinate WNK kinases and KLHL3 in cells, but also was more heavily “NEDD”ylated and activated than wild-type CUL3. In their studies, nephron-specific deletion of *Cul3* in mice increased WNK levels and the abundance of phosphorylated NCC. However, over time, the *Cul3* deletion caused renal dysfunction, including hypochloremic alkalosis, diabetes insipidus and salt-sensitive hypotension, with depletion of NKCC2 and aquaporin 2. Gong *et al* (2015) next reported the identification of claudin-8 as a previously unidentified physiologic target for KLHL3. They provided an alternative explanation for the collecting duct's role in PHA2. They found that deletion of claudin-8 in the collecting duct of mouse kidney caused hypotension, hypokalemia, and metabolic alkalosis, an exact mirror image of PHA2. Responsible for the PHA-2 phenotype was disruption of the claudin-8 interaction with claudin-4, the paracellular chloride channel, and delocalization of claudin-4 from the tight junction. Their findings have added the concept that the paracellular pathway is physiologically regulated through the ubiquitination pathway, and its deregulation may lead to diseases of electrolyte and blood pressure imbalances.

Now, in this issue of *EMBO Molecular Medicine*, Schumacher *et al* (2015) report that the same CUL3Δ403–459 mutation studied by McCormick *et al* (2014) is severely compromised in its ability to ubiquitinate WNKs, possibly due to altered structural flexibility. Instead, CUL3Δ403–459 auto-ubiquitinates and loses interaction with two CUL3 regulators, namely the COP9-Signalosome and cullin-associated and neddylation-dissociated 1 (CAND1). They used a novel knock-in mouse model of CUL3WT/Δ403–459 to recapitulate the human PHA2E phenotype. Their mice also displayed changes in arterial pulse waveform, suggesting a vascular contribution to hypertension not previously appreciated. Their results imply an explanation for the severity of PHA2E phenotype caused by CUL3 mutations compared to mutations in KLHL3 or the WNKs. Particularly exciting is the notion that the hypertension in this model is not only the result of increased sodium reabsorption but also related to an increased contractile state in the vascular tree. Additional studies could be conducted in this model to pursue this possibility further. Interestingly, CUL3 was found to regulate vascular smooth muscle function and blood pressure via PPARγ and RhoA/Rho-kinase in an earlier study (Pelham *et al*, 2012). Furthermore, perivascular supporting cells in the eye, including smooth muscle cell/pericytes, have been investigated regarding proteasome activity. Aghdam *et al* showed that retinal endothelial cells have significantly higher proteasome peptidase activity compared to smooth muscle pericytes. High-glucose treatment increased the level of total ubiquitin-conjugated proteins in cultured retinal smooth muscle pericytes and endothelial cells. Diabetic mice had higher levels of PA28-β/-γ, Cul1 and Cul3 proteins in their intraglomerular capillaries in that study (Aghdam *et al*, 2013). CUL3-related mechanisms in the vasculature that contribute to hypertension appear worthy of pursuit.

hypertension in this model is not only the result of increased sodium reabsorption but also related to an increased contractile state in the vascular tree

To persons outside the field, “it's hard to tell the players without a score card”. A crude attempt at some of the players at least is shown (Fig 1). Suffice it to say that Mendelian forms of hypertension are few in number. Interestingly, at least three syndromes, involving KLHL3, CUL3, and NEDD4, involve failure of proper ubiquitination (garbage disposal). Clear for all to see is what happens when the garbage workers go out on strike! For persons inside the field, matters are no easier. Hypertension features increased blood pressure with normal cardiac output, and therefore, peripheral vascular resistance must be increased (Fig 2). PHA2 clearly occurs inside the kidneys, but how increased NaCl reabsorption leads to increased peripheral vascular resistance is not all that clear. The novel suggestion that the blood vessels themselves could contribute, leading to failed vasodilation at high salt intake, could be very helpful in that regard.

**Figure 1:**
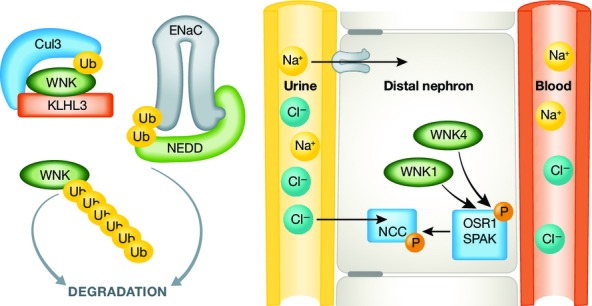
Regulation of WNK signaling by KLHL3–CUL3 complex Under normal conditions, protein levels of WNK1 and WNK4 in DCT are maintained by degradation through ubiquitination by the KLHL3–CUL3 E3 ligase complex. NEDD is responsible for ENaC disposal, also by ubiquitination (left). WNK1 and WNK4 regulate OSR1 and SPAK, which in turn regulate NCC, all for the purpose of sodium and chloride reabsorption.

**Figure 2:**
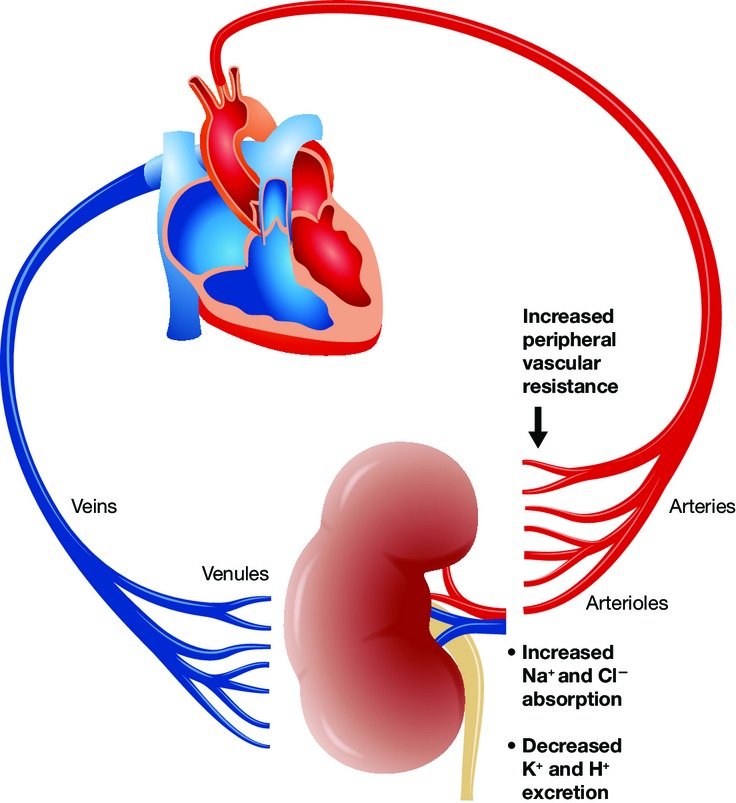
All hypertension requires an increase in peripheral vascular resistance to be sustained In PHA2, NaCl absorption is increased, while K^+^ and H^+^ secretion are impaired. CUL3 mutations impair ubiquitination of WNK. The current findings suggest that the disease may not be merely a renal affair but could directly involve regulation of peripheral vascular resistance.
